# Wearable Digital Sensors to Identify Risks of Postpartum Depression and Personalize Psychological Treatment for Adolescent Mothers: Protocol for a Mixed Methods Exploratory Study in Rural Nepal

**DOI:** 10.2196/14734

**Published:** 2019-09-11

**Authors:** Anubhuti Poudyal, Alastair van Heerden, Ashley Hagaman, Sujen Man Maharjan, Prabin Byanjankar, Prasansa Subba, Brandon A Kohrt

**Affiliations:** 1 Division of Global Mental Health Department of Psychiatry and Behavioral Sciences George Washington School of Medicine and Health Sciences Washington, DC United States; 2 Human and Social Development Human Sciences Research Council Pietermaritzburg South Africa; 3 Medical Research Council/Wits Developmental Pathways for Health Research Unit Department of Paediatrics, Faculty of Health Sciences University of the Witwatersrand Johannesburg South Africa; 4 Department of Social and Behavioral Sciences Yale School of Public Health Yale University New Haven, CT United States; 5 Center for Methods in Implementation and Prevention Science Yale University New Haven, CT United States; 6 Transcultural Psychosocial Organization Nepal Kathmandu Nepal; 7 United Mission to Nepal Kathmandu Nepal

**Keywords:** developing countries, feasibility studies, mobile health, mother-child interaction, postpartum depression, psychotherapy

## Abstract

**Background:**

There is a high prevalence of untreated postpartum depression among adolescent mothers with the greatest gap in services in low- and middle-income countries. Recent studies have demonstrated the potential of nonspecialists to provide mental health services for postpartum depression in these low-resource settings. However, there is inconsistency in short-term and long-term benefits from the interventions. Passive sensing data generated from wearable digital devices can be used to more accurately distinguish which mothers will benefit from psychological services. In addition, wearable digital sensors can be used to passively collect data to personalize care for mothers. Therefore, wearable passive sensing technology has the potential to improve outcomes from psychological treatments for postpartum depression.

**Objective:**

This study will explore the use of wearable digital sensors for two objectives: First, we will pilot test using wearable sensors to generate passive sensing data that distinguish adolescent mothers with depression from those without depression. Second, we will explore how nonspecialists can integrate data from passive sensing technologies to better personalize psychological treatment.

**Methods:**

This study will be conducted in rural Nepal with participatory involvement of adolescent mothers and health care stakeholders through a community advisory board. The first study objective will be addressed by comparing behavioral patterns of adolescent mothers without depression (n=20) and with depression (n=20). The behavioral patterns will be generated by wearable digital devices collecting data in 4 domains: (1) the physical activity of mothers using accelerometer data on mobile phones, (2) the geographic range and routine of mothers using GPS (Global Positioning System) data collected from mobile phones, (3) the time and routine of adolescent mothers with their infants using proximity data collected from Bluetooth beacons, and (4) the verbal stimulation and auditory environment for mothers and infants using episodic audio recordings on mobile phones. For the second objective, the same 4 domains of data will be collected and shared with nonspecialists who are delivering an evidence-based behavioral activation intervention to the depressed adolescent mothers. Over 5 weeks of the intervention, we will document how passive sensing data are used by nonspecialists to personalize the intervention. In addition, qualitative data on feasibility and acceptability of passive data collection will be collected for both objectives.

**Results:**

To date, a community advisory board comprising young women and health workers engaged with adolescent mothers has been established. The study is open for recruitment, and data collection is anticipated to be completed in November 2019.

**Conclusions:**

Integration of passive sensing data in public health and clinical programs for mothers at risk of perinatal mental health problems has the potential to more accurately identify who will benefit from services and increase the effectiveness by personalizing psychological interventions.

**International Registered Report Identifier (IRRID):**

DERR1-10.2196/14734

## Introduction

### Background

There is a global crisis of untreated depression [1]. Lack of treatment for postpartum depression is especially harmful because of long-term consequences for mothers and their children [2]. In low- and middle-income countries (LMICs), prevalence of postpartum depression ranges from 3% to 32% [3]. Among adolescents in South Asia, pregnancy is also a risk factor for suicide [4,5]. Fortunately, there has been development and testing of interventions for perinatal depression delivered by nonspecialists in LMICs [6-8]. However, these have shown limited improvement over control conditions [7,8] and lack of long-term benefits [9].

These issues raise questions including the following: (1) are the mothers most likely to benefit from psychological interventions appropriately identified? and (2) are beneficiaries in the interventions achieving the behavioral changes needed for sustained improvements in mental health? Until recently, we have not had feasible methods for collecting data on the daily lives of depressed adolescent mothers in LMICs and the impact of psychological interventions on their self-care, interpersonal relations, and parenting behaviors. Collection of passive sensing data through devices that mothers already used supplemented with unobtrusive additional wearable technology on children has the potential to shed light onto mothers’ experiences of and recovery from postpartum depression.

Passive sensing data collection is the accumulation of information from digital devices while users go about their daily lives without requiring their active input [10,11]. Examples of passive data collection from mobile phones include Global Positioning System (GPS) location, physical activity and movement, and amount of time that a device or app is used, such as Web-based social activity. Current health applications of passive sensing data collection include recording physical activity among persons at risk for anemia, neurodegenerative diseases, and cardiometabolic disease [12-16].

Passive sensing data collection in mental health may help in correct identification of who will benefit from different types of psychological interventions. At present, the field is limited by the approaches used to identify women with postpartum depression in low-resource settings. Self-report checklists—such as the Edinburgh Postnatal Depression Scale [17] and Patient Health Questionnaire (PHQ-9) [18]—are typically used to identify whom to enroll in an intervention [19]. These screening tools are often used as de facto diagnostic tools in LMIC settings where specialists are not available to provide mental health diagnoses [20]. As screening tools, these self-report measures typically have good sensitivity (ie, they identify most persons in need of care, and there are few false negatives); however, they have low specificity (ie, there are many false positives who get included but do not actually have depression) [21]. The problem of these psychometric properties is even greater when working cross-culturally where tools need to be adapted and validated to the local language, culture, and context [22]. One of the reasons for small effect sizes in psychological treatment trials may be that these approaches lead to the inclusion of women with modest distress who would naturally recover in research trials.

Passive sensing data can potentially be used to increase the accuracy of detecting women with postpartum depression who would benefit from psychological interventions. For mental illness, passive sensing data can be used to create digital phenotypes related to activity patterns, sleep, social interactions, and emotional tone of speech [10,23-25]. This approach has shown promise in other neuropsychiatric disorders. An example includes use of passive sensing data to record motion density of activity, circadian rhythm, time away from home, and activity level to identify older adults with risk of early dementia [12]. Similar digital phenotypes are likely to inform postpartum depression risk assessment [26,27].

**Figure 1:**
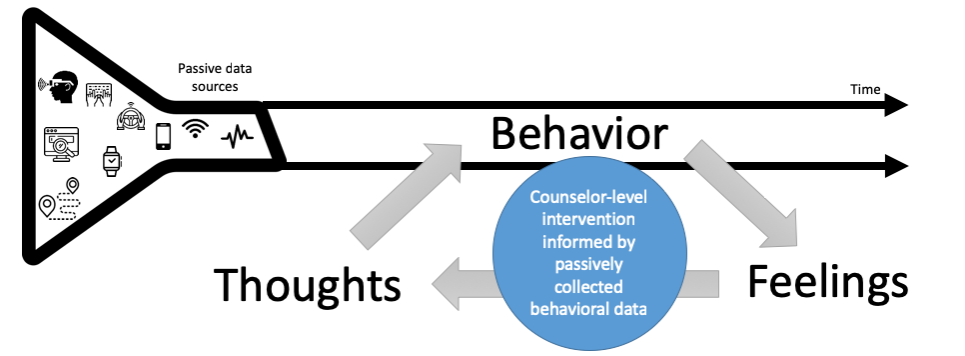
Model illustrating the role of passively collected behavioral data to enhance behavioral change among treatment beneficiaries in psychological interventions based on behavioral activation and cognitive behavioral techniques.

In addition to helping with diagnosis and symptom monitoring, passive sensing data collection also can be used to improve interventions. For example, monitoring physical activity and then providing positive reinforcement when activity milestones are achieved has been used for diabetes risk reduction and treatment programs [15]. Similarly, the information garnered through digital phenotyping has the potential to improve the delivery of mental health interventions [28]. In Figure 1, we illustrate how passive sensing data collection from wearable devices can be used to generate behavioral profiles over time.

Intervention providers who are nonspecialists, such as trained lay counselors, could then use the behavioral information when interacting with treatment beneficiaries to explore cognitive and emotional processes (thoughts and feelings) associated with depression. Using behavioral activation or cognitive behavioral techniques, the provider and treatment beneficiary can then collaboratively plan goals for changes in behavior, thoughts, and feelings, with the former monitored through ongoing passive data collection.

Given the need to improve accurate detection of depression through passive sensing data and the potential to incorporate passive sensing data to enhance mental health care, we undertook an exploratory study with wearable digital sensors used by adolescent mothers and their infants in rural Nepal.

### Objectives

Our study has 2 objectives. First, we will explore the feasibility and acceptability of passive sensing data collection among depressed and nondepressed adolescent mothers with infants (aged <12 months) and conduct exploratory analyses comparing these data in 4 domains: (1) the overall activity of mothers using accelerometer data from the mobile phones, (2) the geographic range and routine of mothers using GPS data collected from the mobile phones, (3) the time and routine of mothers with their infants using proximity data collected from Bluetooth beacons, and (4) the verbal stimulation and overall auditory environment for mothers and infants using episodic audio recording on mobile phones. Second, we will explore the feasibility and acceptability of collecting passive sensing data from depressed adolescent mothers who are participating in a psychological intervention (behavioral activation) delivered by nonspecialists. These data will then be used to contextualize and personalize the psychological intervention. During the 5 weeks of the psychological intervention, we will document how the passive sensing data are used by the intervention providers in personalizing the intervention, and we will conduct exploratory analyses on changes in the 4 domains over time.

In this study protocol, we describe the global health research setting (Nepal), the psychological intervention (an adaptation of behavioral activation), the technology to be used (a mobile phone given to adolescent mothers and a passive Bluetooth beacon attached to their infants’ clothing), the mobile phone app we have developed (which intervention providers can use to integrate passive sensing data into the psychological intervention), and the study components (a participatory research element, a mixed-methods comparison of depressed and nondepressed mothers, and a mixed-methods evaluation of passive sensing data integrated into psychological care).

## Methods

### Setting

Nepal is one of the poorest countries in South Asia with an economy that relies heavily on remittance from migrant laborers, development aid from high-income countries, and tourism. Nepal has a population of approximately 26.4 million, with 69.1 years life expectancy at birth. The United Nations ranks Nepal 145th out of the world’s 188 countries on the Human Development Index, indicating that Nepal has lower life expectancy, education level, and per capita income when compared with other countries [29]. This study will be conducted in Chitwan, a southern district bordering India. The total population of Chitwan is 579,984 (300,897 females) with about 132,462 households [30,31]. Chitwan has a slightly better health and development indicators than the national average. The infant mortality rate is 30.1 per 1000, lower than the national average of 40.5 per 1000. The under-five mortality rates for Chitwan is 38.6 per 1000 (national average is 52.9). Chitwan also has a higher literacy rate than the national average—78.9% in Chitwan compared with the national average of 67% [32].

The estimated age-standardized suicide rate in Nepal is the eighth highest in the world, with the female suicide rate ranking the third highest [33]. Suicide is a leading cause of deaths among reproductive-aged women in Nepal [34-36], with the highest rates of suicide among women younger than 25 years of age [37]. In Nepal, young mothers are burdened with persistent gender inequity and disproportionate expectations often placed on them following marriage [38-40]. Patrilocal tradition typically requires women to leave their maternal home and assume a relatively low social position in their husbands’ households. This can be socially isolating and accompanied by increased exposure to violence [40]. During periods of menstruation, childbirth, and early marriage, some families practice restrictions on women, limiting their geographic movements, physical and social interactions, and religious practices [41]. Social and psychological benefits for women, including upward social mobility, increased fulfillment and life satisfaction, and generativity, are typically contingent upon becoming a mother and, more specifically, giving birth to a son [42,43]. Given the cultural milieu, it is possible that passive sensing technology may help to identify behavioral opportunities to mitigate the risk factors, such as social isolation, associated with adolescent motherhood.

In Nepal, mental health services are restricted to a few government hospitals located in big cities and private hospitals. In Chitwan, mental health services include inpatient and outpatient services available in the district hospital and medical colleges. With 2 psychiatrists and a psychiatric ward in the district public hospital, the district has more capacity compared with most areas in Nepal. Over the past 8 years, Chitwan has been the Nepal implementation district for the Program for Improving Mental Health Care (PRIME), which has contributed to an increase in availability of mental health services. On the basis of PRIME studies, 12-month prevalence of suicidal ideation is 3.5% and attempts are 0.7% in community settings and 11.2% for ideation and 1.2% for attempts among patients presenting to primary care [44]. Moreover, in primary care settings, 11.2% of attendees were found to have depression, but only 1.8% had sought mental health services in primary care [45]. A psychological treatment for postpartum depression was adapted for use in Chitwan through the PRIME activities (additional details are provided in the Intervention section).

Regarding the use and availability of mobile digital technologies, there has been a drastic increase in mobile penetration in the last decade. There are currently 2 leading telecommunication companies in Nepal, along with a few smaller mobile service providers. The nationwide mobile penetration in 2018 was 134% [46]; this number of mobile service plans per person is greater than 1 because many individuals have 2 or more plans with different service providers due to differences in coverage networks.

### Intervention

The psychological intervention used in this study is the Healthy Activity Program (HAP) [45]. Although other low-intensity psychological interventions (eg, the Thinking Healthy Program) [6] have been developed for use in South Asia to treat postpartum depression, HAP has shown strong benefit with general depression treatment in India [47] and has added benefit for depression when combined with primary care treatment in Nepal [48]. In addition, HAP has similar psychological elements to the Thinking Healthy Program, with behavioral activation being a major component [47]. Behavioral activation is a type of psychological treatment developed out of cognitive therapy, grounded in learning theory; it has 2 primary components: the use of avoided activities as a guide for activity scheduling and the functional analysis of cognitive processes that involve avoidance [49]. Simplified versions, such as most variants used by nonspecialist providers in LMIC, emphasize the activity scheduling more than the functional analysis.

Through PRIME in Nepal, HAP has been adapted for perinatal depression, and there are numerous government health workers trained in HAP for perinatal depression in Chitwan, Nepal. The HAP intervention that is currently being implemented in Nepal has been divided into 3 phases delivered over 5 sessions (see Textbox 1). Although HAP includes both behavioral activation and problem-solving therapy techniques, the behavioral activation element is the component in which passive sensing data are integrated for this study.

**Phases and sessions of the Healthy Activity Program adapted for use in Nepal.**

**Phase I: Assessment and psychoeducation**

Session 1: Initial assessments for psychosocial well-being and self-care

1. Assurance of confidentiality
2. Assessment of depression risk through the administration of a validated screening tool—Beck Depression Inventory (BDI).
3. Assessment of suicidal risk
4. Assessment of 6 components of self-care (work, rest, nutrition, interpersonal relationships, entertainment, and health)
5. Discussion on the possibility of involvement of family in the Healthy Activity Program (HAP)
6. Homework and discussion on the possible barriers for completion
7. Planning for the next session

Session 2: Psychoeducation based on HAP and self-care

1. Progress review based on BDI and self-care
2. Assessment of suicidal risk
3. Review homework
4. Psychoeducation on HAP and self-care
5. Selection of the activity during the session
6. Involvement of the family member
7. Homework and discussion on the possible barriers for completion
8. Planning for the next session

**Phase II: Behavioral activation and problem solving**

Sessions 3 and 4: Behavioral activation and problem solving

1. Progress review based on BDI and self-care
2. Assessment of suicidal risk
3. Review homework
4. Problem-solving methods
5. Involvement of a family member
6. Homework and discussion on the possible barriers for completion
7. Planning for the next session

**Phase III: Relapse prevention and wrap up**

Session 5: Relapse prevention and wrap up

1. Progress review based on BDI and self-care
2. Assessment of suicidal risk
3. Review of skills learned
4. Involvement of a family member
5. Relapse prevention
6. Debrief and wrap up

These sessions have been designed based on HAP as delivered by nonspecialists in LMIC settings [50]. Broadly, each HAP phase has the following content:

#### Early Phase (Delivered in 1-2 Sessions)

In this phase, the psychosocial counselor engages with the participant and establishes an effective counseling relationship. It also involves describing HAP to the patients and eliciting participant’s commitment to continue and complete the counseling sessions.

#### Middle Phase (Delivered in 3-4 Sessions)

In this phase, the counselor assesses behavioral activation targets and encourages positive behaviors. The counselor, along with the participant, identifies the barriers to activation and ways to overcome these barriers. This phase also involves helping patients solve or cope with life problems.

#### Ending Phase (Delivered in 1 Session)

In this phase, the counselor reviews the progress in the last few weeks and discusses with the patient ways to strengthen the gains to prevent relapse.

At present, 20 health facilities in Chitwan have adapted HAP for the treatment of maternal depression. In these settings, HAP is delivered by auxiliary nurse midwives who are part of the formal paid health infrastructure in Nepal. Auxiliary nurse midwives receive 18 months of training after a high school degree that is focused on midwifery, reproductive health including family planning, and community health. For HAP, auxiliary nurse midwives receive 5 days of training on basic psychosocial skills. Those displaying the strongest competency (as evaluated through observed structured role plays) [51] and those with good knowledge and attitudes then participate in 5 days of HAP training. After training, the auxiliary nurse midwives receive in-person supervision from a psychosocial counselor who has completed a 6-month training specialized for Nepal [52]. HAP supervision from psychosocial counselors (and psychiatrists when necessary) occurs approximately biweekly with additional phone and in-person support as needed. The psychosocial counselor supervisors also provide HAP services in areas without trained midwives. For this study, we will include depressed adolescent mothers who are receiving HAP from either an auxiliary nurse midwife or a psychosocial counselor (collectively referred to as *providers* in this protocol).

### Conceptual Model

To guide our study, we developed a simple conceptual model (see Table 1) demonstrating how the passive sensing data domains may distinguish differences between depressed and nondepressed adolescent mothers (objective 1) and how domains could be integrated into a brief behavioral activation psychological intervention (objective 2).

**Table 1:** Conceptual domains related to depression that can be monitored through passive sensing data collection.

Domain	Description	Association with depression	Passive sensing data	Use of passive sensing in the psychological intervention
Physical activity	Time spent inactive, standing, walking, or riding vehicles	Lack of physical activity associated with depression	Accelerometer data from mobile phone provided to mother	Determine targets for physical activity and monitor changes in type and duration of physical activity
Geographic movement	Range and location of daily movement in community	Lack of daily movement outside the home and lack of routine in movement associated with depression	GPS^a^ data from mobile phone provided to mother	Identify locations for mood-enhancing activities and monitor movement to those settings
Mother-child interaction	Total time of mother and child together and daily consistency of mother-child routine	Lack of mother’s time separate from child (ie, no break from child care responsibilities) and inconsistency of daily routine (ie, erratic schedule) associated with depression	Mother-child proximity measured between mobile phone with mother and passive Bluetooth beacon attached to child’s clothing	Identification of times for mood-enhancing activities with and without child and monitoring to increase consistency of daily routine
Interpersonal relations	Exposure to adult verbal communication and verbal communication of child	Lack of exposure to adult verbal communication and lack of verbal engagement with child associated with depression	Episodic audio recordings collected on mobile phone given to mother	Determining targets for social interaction and monitoring adult communication and verbal stimulation of child

^a^GPS: Global Positioning System.

#### Domain 1: Daily Routine of Physical Activity of Mothers

The Android operating system provides access to several sensors that enable the monitoring of motion. For this study, we used the accelerometer and gyroscope sensors along with the Activity Recognition API, which is built on top of these sensors. The Activity Recognition API automatically detects activities such as walking, riding in a vehicle, and standing. It does this by passing these sensor data into a machine learning model. These data will be interpreted along with GPS data to estimate the frequency, quantity, and type of activity undertaken. We hypothesize that depressed mothers will have less physical activity and less consistent routine of physical activity compared with nondepressed mothers. Self-reported physical limitations are correlated with postpartum depression severity [53]. Prospective studies of women during pregnancy and the postpartum period using self-report measures of daily rhythms demonstrated that women with disrupted sleep and daily rhythms had worsening of depressive symptoms [54]. In the same study, women with histories of mood disorder were more likely to report disrupted rhythms. Wrist actigraphy measurements among postpartum women also showed an association between disrupted routines and poor mental health outcomes; postpartum women with dysrhythmic fatigue patterns reported more stress and less vigor compared with the women where fatigue patterns followed consistent daily cycles [55]. Moreover, clinical insomnia is associated with less regularity in daily physical activity [56]. Therefore, for our first objective, we will explore if women without depression have more stable activity routines compared with women with postpartum depression. For our second objective, we will explore how providers use the activity data to identify and monitor mood-enhancing physical activity. We hypothesize that depressed mothers in the intervention will increase their physical activity and the routinization of their physical activity over the course of the intervention. This would be expected because 1 element of behavioral activation included in HAP is the scheduling of behaviors that have mood-enhancing qualities.

#### Domain 2: Geographic Range and Location of Mothers’ Routine

This domain will use GPS data collected from a mobile phone. In a study of pregnant mothers, the daily radius of travel was associated with depression symptoms, with greater depression levels associated with more restricted radii of travel [57]. The same study also found that an increase in depressive symptoms predicted smaller radii of travel in subsequent days. For objective 1, we hypothesize that mothers with depression will have more restricted GPS range of movement compared with nondepressed mothers, that is, they will be more isolated and show less movement outside the home. For objective 2, we will explore how providers delivering HAP use the GPS data to identify targets for increased social engagement and physical activity, as well as monitor engagement in mood-enhancing locations identified by the depressed mothers. We hypothesize that depressed mothers in the intervention will show increased GPS geographic range over the course of the intervention.

#### Domain 3: Duration and Routine of Mothers’ Interaction With Their Infants

This information will be generated by a Bluetooth beacon (RadBeacon Dot; Radius Networks, Inc) [58] attached to the child’s clothing. Every 15 min, the phone will scan for the presence of the beacon and determine the distance between the devices (proxies for the individuals) using received signal strength indication (RSSI). If the beacon is not detected, the mother and child are assumed to be apart. These data will be used to determine the total amount of time a mother and child spend together each day and the routine of their time together. As mentioned above, routinization of daily behaviors is associated with more positive mood, less fatigue, and lower risk of maternal depression [54,55]. Therefore, in addition to physical routine, we will also capture the daily interaction routine between mothers and infants. We hypothesize that the nondepressed mothers in the study are likely to have more consistent routines over the 2-week period compared with depressed mothers. We also hypothesize that depressed mothers will have less time apart from their child (eg, have no break from child care responsibilities). Self-reported lack of social support for child care activities is correlated with risk and severity of postpartum depression [53,59,60]. High levels of instrumental social support are associated with lower postpartum depression symptom severity [61]. Among low-income Latina women in the United States, lack of partner engagement was associated with greater childcare responsibilities and greater risk of maternal depression [62]. A study in Kenya found that providing social support and addressing caregiver burden were especially important for pregnant adolescents [63]. With regard to the mothers in the psychological treatment, we will explore if providers use the daily proximity data to identify opportunities for mood-enhancing activities. We also hypothesize that mothers will show increasing routinization of their schedule with the child over the course of the intervention.

#### Domain 4: Verbal Stimulation and Auditory Environment for Mothers and Infants

Using episodic audio recording on a mobile phone, 30 seconds of audio will be recorded every 15 min on the mobile phone provided to the mother. We hypothesize that depressed mothers are more likely to have prolonged periods without verbal communication compared with nondepressed mothers (ie, depressed mothers will have greater silence throughout the day). Audio recordings with human speech will be used as proxy for social interaction. Social isolation is associated with postpartum depression [64]. Loss of social group membership is a risk factor for postpartum depression [65], and this loss of social group is of particular risk for adolescent mothers. In addition, limited verbal engagement between mothers and infants is a manifestation of postnatal depression and predicts poor development for children [66,67]. For objective 2, we hypothesize that depressed mothers in the psychological intervention will show increasing exposure to verbal communication over the course of the intervention. Changes in loneliness and perceived social support are associated with the course of postpartum depression [68]. Women with perinatal depression in therapy showed reduction in depression associated with greater interaction with their infants [69].

### Technology

For the project, we are using 2 devices—a mobile phone (Samsung J2 Ace) and a passive Bluetooth beacon (RadBeacon Dot) paired with the phone to serve as a proximity sensor.

#### Mobile Phone

The Samsung J2 Ace phone is a cost-effective mobile phone (US $114) that is popular in the study setting. Commonly-used low-end mobile phones in Nepal cost US $70-US $120, and therefore the device selected the study was only modestly more expensive than commonly-used devices. Most individuals in the area already own a mobile phone or have a close family member with a mobile phone. Hence, there is minimum risk of stigmatization because of the mobile phone use in the study. We selected the Samsung J2 Ace phone because it is widely available for purchase within Nepal and it was the cheapest option that could effectively run all the features and apps required for the study. The participants will be provided with the phone for the duration of the study. They will return the phone after the data collection. This information is clearly stated in the consent form.

The mobile phone will be used to collect 4 types of data—proximity, episodic audio, activity, and location. To collect these data, we will install our custom-built Electronic Behavior Monitoring app (EBM version 2.0). The EBM app is designed to passively collect data for 30 seconds every 15 min between 4 am and 9 pm. First, the EBM app scans for the presence of advertising packets from the assigned Bluetooth beacon. For episodic audio recording, the microphone in the phone will be used to record 30-second audio clips saved in an MP3 format. The audio data will be collected directly on the mobile phone and uploaded in our cloud-based storage. The processing service that uses machine learning model then converts the audio into a categorical variable with a confidence score; therefore, the research staff and counselors never hear the audio. We produced a video to explain this process to participants, which was published in a previous paper [70]. Moreover, the participants can request for audio files to be deleted before uploading. Participants can also turn off their phone anytime. We will make the participants aware of these options. We have piloted the approach with participants collecting the audio and deleting it in South Africa [71]. Finally, GPS on the mobile phone will collect the mother’s position, and the Activity Recognition API used to record the predicted activity being undertaken at the time of recording. A folder, NAMASTE, is created automatically once the EBM app is downloaded on the mobile phone. All data are recorded within the folder.

#### Passive Bluetooth Beacon (RadBeacon Dot) (Radius Networks Inc)

**Figure 2:**
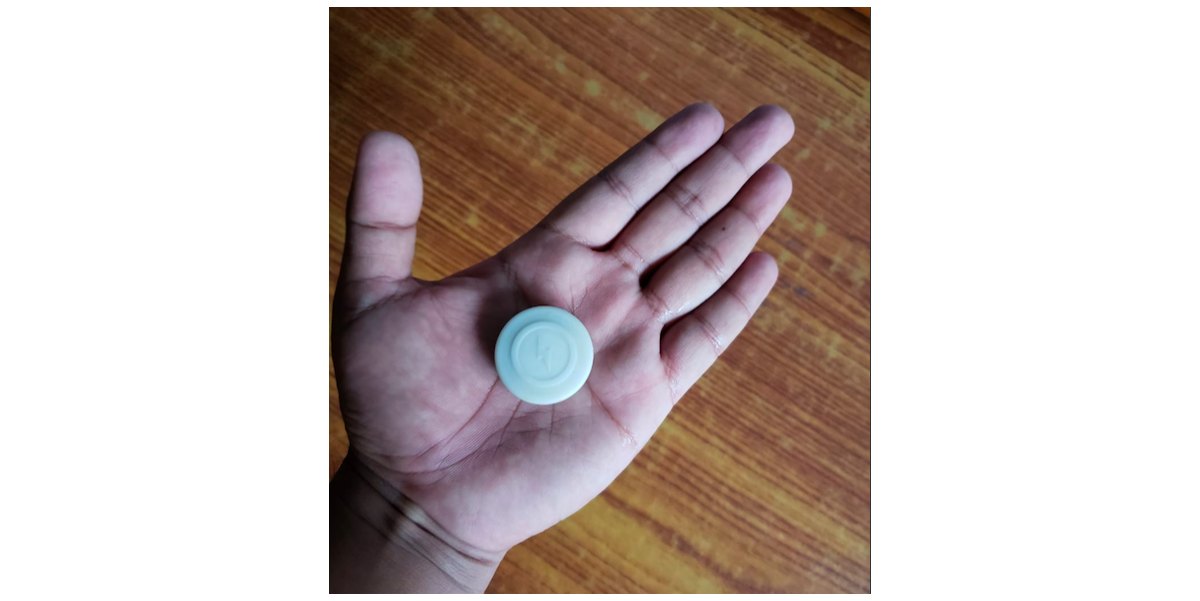
RadBeacon Dot (Radius Networks Inc.).

The proximity sensor (Figure 2) is fitted to the child’s clothing, and the mother is asked to carry the mobile phone to measure the distance between the mother and child. Our assumption is that the beacon is always on the child, and the mobile phone is with the mother. The EBM app will scan for beacons and record proximity information every 15 min, which will give an indication of how often the mother and the child are physically close. In addition, the RSSI gives an approximation of the distance between mother and child. The RadBeacon transmission power was set to show the child as *in proximity* if the distance between phone and beacon was less than 7 m [72]. This distance is affected by the presence of walls, furniture, and other obstacles between the mother and child.

Use of the RadBeacon Dot was approved by the Nepal Health Research Council for the purpose of this study. RadBeacon Dot also has United States Federal Communications Commission (FCC) certification, a body that oversees the permissible exposure level for all devices with radio frequency. In the United States, the Food and Drug Administration (FDA) relies on FCC for inputs on medical devices [73]. FDA considers devices such as activity trackers as general wellness devices because these devices have “(1) an intended use that relates to maintaining or encouraging a general state of health or a healthy activity or (2) an intended use that relates the role of healthy lifestyle with helping to reduce the risk or impact of certain chronic diseases or conditions and where it is well understood and accepted that healthy lifestyle choices may play an important role in health outcomes for the disease or condition” [74].

Moreover, in our study, RadBeacon is not a medical device used for treatment or for the transmission of health information (eg., temperature, pulse, respiration) from the infant. It is only used to track proximity between mother and infant during daytime hours. Regarding the safety of exposure for infants, the FCC limit for radiation from devices is 1600 mW/kg [75], which equates to approximately 800 mW for a 5 kg infant. The RadBeacon Dot specifications are +4 to −20 dBm, which equates to 2.5 to 0.1 mW. These ranges are comparable to an infant in a house with a standard wireless network and Bluetooth devices.

#### Preliminary Studies of These Technologies in Nepal

We conducted preliminary studies on the collection of passive sensing data in rural Nepal [70]. We developed videos demonstrating the use of passive sensing data collection in households in Nepal. The videos featured devices for continuous video recording, continuous audio recording, episodic audio recording, as well as wearable cameras placed on the children, Bluetooth beacons placed on the children, and environmental sensors in the home. The videos were shown to female community health volunteers and mothers with young children to assess their perspectives on how passive data collection impacted confidentiality, safety of their children, social acceptability in the family and community, and level of interference on daily activities. In addition, we asked community health volunteers and mothers to identify which types of passive data collection devices they considered most likely to have utility for improving child development and maternal-child interactions. In rural Nepal, the use of passive Bluetooth beacons placed on the children to monitor when the child was in proximity to the caregiver scored well on these criteria, especially confidentiality and social acceptability. Episodic audio recording had similar perceived utility and social acceptability, but caregivers wanted to assure that confidentiality could be maintained. Regarding safety and low risk of interference in daily life, the episodic audio recording scored better than the proximity beacon. A total of 3 devices (proximity beacon, episodic audio recording, and a child’s wearable camera) were then piloted with mothers and children aged 2 to 5 years. On the basis of the results of that pilot study (unpublished data), we selected the proximity beacon and episodic audio recording as appropriate for mothers and their infants aged younger than 1 year in this study.

### The StandStrong App

The Sensing Technologies for Maternal Depression Treatment in Low Resource Settings (StandStrong) Platform will be an Android App that the providers can use to access the passive sensing data about participants in HAP. Through this platform, the counselor can review the data collected by the EBM app and provide personalized HAP sessions to the mothers. Additional functionality will be direct text messaging between the mother and the providers, summary of HAP sessions, awards and goals for mothers (contingent to behavioral activation), and psychoeducational materials.

We designed the StandStrong app to compliment HAP. This was not a new intervention that we developed, as HAP was already developed for the treatment of depression in South Asia. However, there was not an app to monitor passive sensing data for incorporation into HAP; this was the purpose of developing the StandStrong App. Moreover, we designed the StandStrong app specifically with the conditions of low-resource settings in mind. The app has low data usage, can be used in older versions of Android, and is translatable into various languages. To the best of our knowledge, this is the first app to bring together this specific package of passive data collection and intervention support in the same platform.

**Figure 3:**
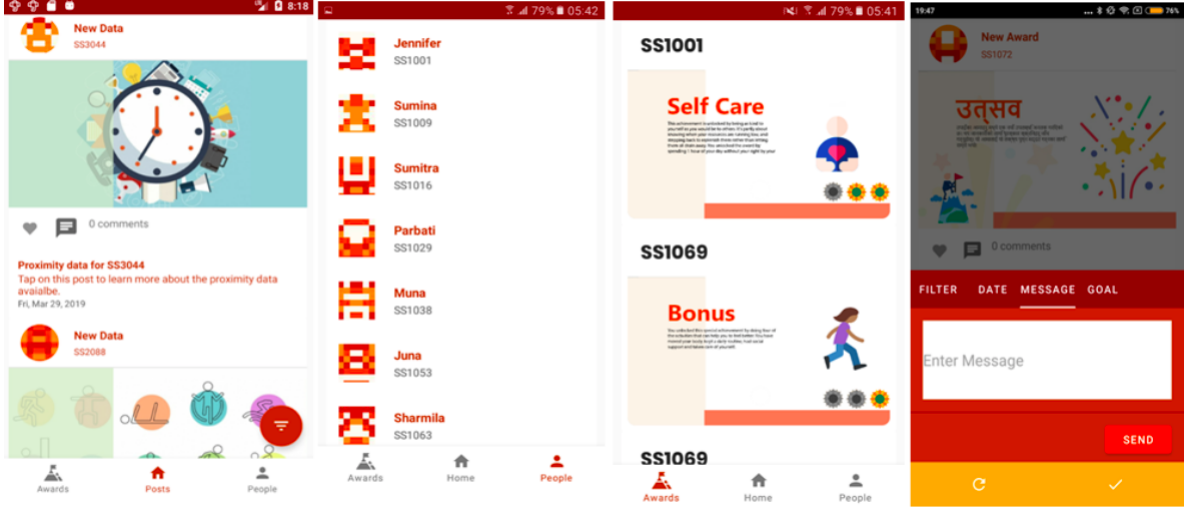
The StandStrong app. From left to right: Homepage, People, Awards, and Direct messaging.

The app has the following 3 main screens (demonstrated in Multimedia Appendix 1):

- Home Page: the homepage works as the newsfeed and provides a list of posts designed to visualize the passively collected data. These posts include proximity, activity, GPS movement, and proximity/daily routine. The counselor will receive a notification when new data are available for the depressed mothers. Moreover, 2 additional posts types are also available that do not rely on passive data. The first is 5 educational posts that are released at a rate of 1 every 3 days. Second, it is possible for the provider to create goals with the client. These can then be reviewed at follow-up visits (Figure 3). All data can be filtered by date and post type.
- Awards: to provide meaningful feedback and encourage positive health behaviors, passive data are automatically reviewed for the attainment of predefined levels of activity. Each of the award categories (Table 2) have 3 levels of attainment that are increasingly challenging to achieve. For example, to achieve level 1 of self-care, the mother needs to spend 1 hour in self-care with another family member or someone else caring for her child. These thresholds were not based on theory or evidence but rather designed to primarily be easily achievable. As data are collected through this pilot study, better calibration of the award levels will be performed. Examples of how these awards are displayed in the app can be seen in Figure 3. A mother will receive an award when the passive data meet the threshold for one of the award categories. The provider is able to see these achievements for all of her clients in the StandStrong app, and the mother is notified of her achievement through an automated message sent to her Viber account (a popular messaging platform in Nepal).
- People: on the People page, the counselor will see a list of all her active clients (Figure 3). This page allows the counselor to enter the client’s personal page that has all the available data (awards, proximity, activity, and GPS data).

**Table 2:** Award categories.

Award category and level			Description
**Self-care**			
	Level 1	Spend 1 hour without child on a single day (proximity)	
	Level 2	Spend 1 hour without child for 2 consecutive days (proximity)	
	Level 3	Spend 1 hour without child for 4 consecutive days (proximity)	
**Social support**			
	Level 1	Hear talking at least 2 times (per 15-min recording) in a single day (audio)	
	Level 2	Hear talking at least 4 times (per 15-min recording) in a single day (audio)	
	Level 3	Hear talking at least 6 times (per 15-min recording) in a single day (audio)	
**Routine**			
	Level 1	Similar (1-hour variance) pattern of proximity 2 days in a row (proximity)^a^	
	Level 2	Similar (1-hour variance) pattern of proximity 3 days in a row (proximity)	
	Level 3	Similar (1-hour variance) pattern of proximity 4 days in a row (proximity)	
**Movement**			
	Level 1	Activity other than tilt, sit or still 1 time in a day (accelerometer)	
	Level 2	Activity other than tilt, sit or still 2 times in a day (accelerometer)	
	Level 3	Activity other than tilt, sit or still 3 time in a day (accelerometer)	
**Bonus**			
	Level 1	L1 for all of the above	
	Level 2	L2 for all of the above	
	Level 3	L3 for all of the above	

^a^Daily routine was established by looking at the hourly pattern of time spent together with the child and alone. If the pattern was similar across 2 or more days, the award was triggered. *Similar* was defined as the same state (together or alone) appearing 1 hour before, at the same time, or 1 hour later.

#### Educational Messages

Educational messages, designed to facilitate discussion between providers and mothers, are available within the StandStrong app. Providers can show the messages displayed in the StandStrong app and discuss them with the mothers. The educational messages cover a range of topics such as general depression, perinatal depression, self-care, and sleep regulation. These messages are included in the app with the purpose of psychoeducation for mothers and their family members. It allows participants to learn on their own, as well as discuss these topics in HAP sessions with the counselor.

#### Direct Messaging

The provider can send direct messages to the participants through this feature. Providers use this feature in the app to type out a message, which is then sent to the mother and received on her phone through the Viber app. The participant can respond directly through Viber, and the counselor will receive the message on the StandStrong app (Figure 3).

#### StandStrong Architecture

**Figure 4:**
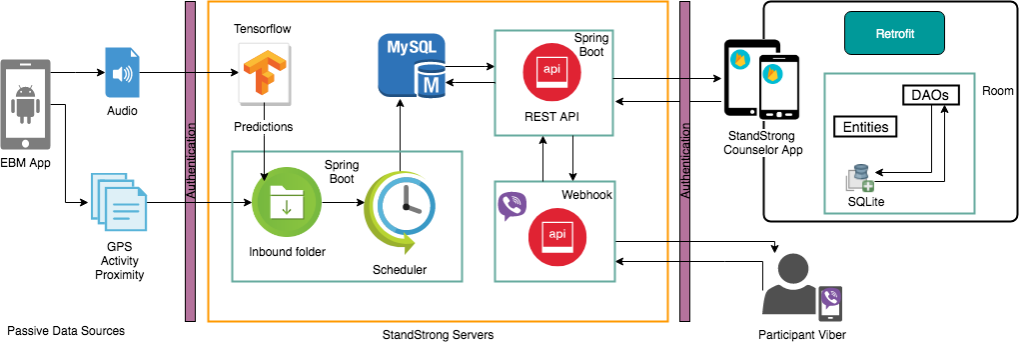
The StandStrong architecture. DAO: Data Access Object; EBM: Electronic Behavior Monitoring; GPS: Global Positioning System; API: Application Programming Interface.

The EBM app captures GPS, activity, and proximity data into text files, which are then loaded into the MySQL database through Scheduler. The captured audio .mp3 files need to be processed through Tensorflow, which predicts the social interaction. Thus, produced predictions are loaded into database through Scheduler. The counselor requires the StandStrong counselor app in offline mode while visiting the participant at home where internet is not available. The app gets synchronized to new data when available in the server through REST API. Using Viber API, the counselor and the participants can post or send messages to each other. Figure 4 shows the StandStrong architecture.

### Study Design

**Figure 5:**
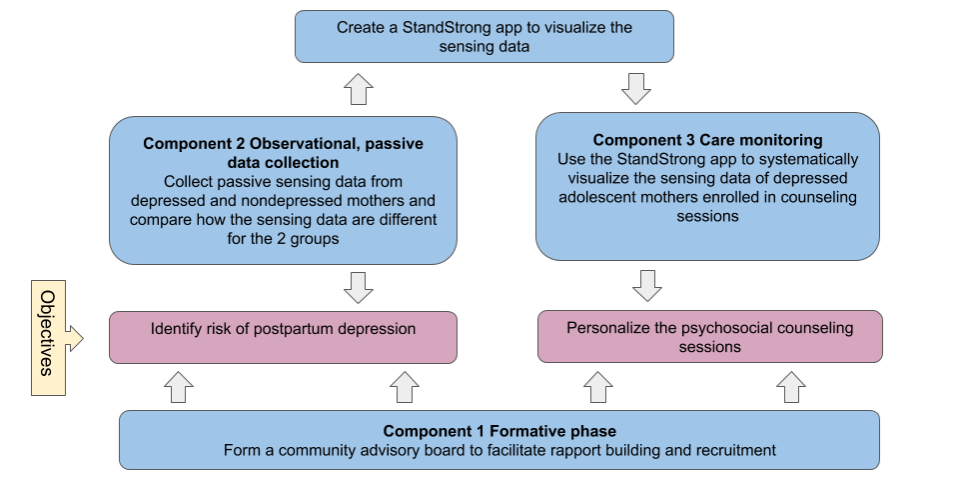
Conceptual map of the study.

The study will be divided into 3 overlapping components (see Figure 5): a qualitative formative component; an observational, passive data collection component; and a care monitoring component. Adolescent mothers (aged 15-25 years) with infants (<12 months) will be recruited for the study. This pilot study will be conducted among participants who will be visiting health facilities in Chitwan, Nepal.

- Component 1: in the formative component, female community health volunteers and auxiliary nurse midwives from 7 health facilities will be invited as the community advisory board members. The community advisory board will facilitate rapport building and networking in health facilities along with inputs on participant recruitment.
- Component 2: employing the recommendations from the community advisory board, we will collect passive sensing data from depressed and nondepressed mothers and compare the data. We will also develop the StandStrong app that will be used to visualize the passive sensing data.
- Component 3: the final component will be the care monitoring phase where we will use the StandStrong app to systematically visualize the passive sensing data of depressed adolescent mothers enrolled in counseling sessions. The providers will have access to the passive sensing data, which can then be used to provide tailored counseling sessions to the mothers.

#### Component 1

In the formative phase, the community advisory board will be established to identify the feasibility and acceptability of using sensing technology among adolescent and young mothers in the community. In addition, community advisory board members will assist in understanding the cultural context, particularly, the experiences of adolescent mothers in the community and the use of mobile technology among young mothers.

##### Participants

The community advisory board will include auxiliary nurse midwives and female community health volunteers currently working in the 7 health facilities. They were included on the basis of the following criteria:

- Preferably aged younger than 30 years.
- Working in the same community for the past 2 years at least.
- Motivated and working with HAP, or experience working on similar mobile health (mHealth) projects

Throughout the project, we will also recruit adolescent mothers to join the community advisory board after they have completed the study procedures.

##### Data Collection

The community advisory board meetings, facilitated by the research staff, will be held at regular intervals throughout the study period to understand perceived challenges for adolescent mothers and the potential for using technology to improve mental health outcomes. In addition, we will elicit feedback throughout the iterative development of the StandStrong app. Focus group discussion data will be collected during the first community advisory board meeting, along with field notes and meeting minutes from each of consecutive meetings. These data will be used alongside the other data in components 2 and 3 to assess the overall feasibility and appropriateness of the platform and study.

#### Component 2

Following the formative phase, we will collect passive sensing data from both depressed mothers (n=20) and nondepressed mothers (n=20) to assess the differences in passive data between these groups. We will also determine the feasibility, acceptability, and utility of the data based on the feedback from the adolescent mothers and community advisory board members.

##### Participants

For the observational passive sensing data phase, female research assistants will reach out to the adolescent mothers in postnatal clinics and immunization camps and then, on receiving consent, administer Patient Health Questionnaire (PHQ-9) for screening. Mothers between the age of 15 and 25 years with infants up to 12 months of age will be considered for recruitment. Research assistants will make home visits for the mothers who agree to participate. During the time, research assistants will discuss the technology and get family consent, which is considered integral in the cultural context. For adolescent mothers aged younger than 18 years, the parental permission form will also be signed at this time. Women with a PHQ-9 score higher than or equal to 10 will be recruited as depressed mothers and those below 7 or equal as nondepressed mothers. We will collect passive sensing data from both depressed and nondepressed mothers for 2 weeks. We will compare the data for these 2 groups.

##### Quantitative Data Collection

A series of mental health and other assessment measures will be used for monitoring mental health and triangulation with sensing data. All tools underwent a standardized process of transcultural translation and validation that our team has used extensively in Nepal [76-79]. This procedure involves producing a Nepali translation, followed by review with Nepali mental health experts. Tools are then evaluated through focus group discussions with Nepali beneficiary populations. A back translation is then reviewed by the study team to compare with the original tool. At each stage, comprehensibility, acceptability, relevance, and completeness of tools are evaluated to determine cultural equivalence. This optimizes semantic, content, construct, and technical equivalence of the items. This assures that somatic complaints, terminology for suicide and self-harm, and idioms of distress are culturally relevant. We also include specific Nepali cultural concepts of distress, such as *heart-mind problems* and ethnopsychological models that are more culturally acceptable to discuss than stigmatized psychiatric terminology [80-83].

1. PHQ-9: the PHQ-9 measures depression symptom severity with 9 items, each with 3 response options and a score range from 0 to 27. It will be administered at the time of study screening. From the validation study in primary care settings in Chitwan, a cutoff score of 10 or more had 94% sensitivity and 80% specificity, and internal consistency of alpha of .84 [80].
2. Beck Depression Inventory (BDI): the BDI is a clinical screening tool used for depression and consists of 21 self-reported items each scoring 0 to 3, with a maximum score of 63. The tool has been validated in Nepal using the local Nepali language among clinical and community participants in urban and rural settings with a cutoff score of 20 [78]. The BDI is also routinely used in clinical practice in Nepal as a measure of symptom improvement. The area under the curve that captures the amount of correctly classified persons in this case for moderate depression was 0.919 (95% CI 0.878-0.960) for the BDI; internal reliability was also high, BDI Cronbach alpha=.90. On the basis of clinical validation of the BDI in Nepal, a score of 20 or higher suggests moderate depression with the need for mental health intervention (sensitivity=0.73 and specificity=0.91). The two-week test-retest reliability Spearman-Brown coefficient for the BDI was 0.84 [84].
3. World Health Organization Disability Assessment Scale (WHODAS): the WHODAS 2.0 12-item tool measures difficulty in daily functioning, including self-care and home activities. Items are scored on a Likert scale from 1 (*none*) to 5 (*extreme/cannot do*), with possible range from 0 to 48 where a higher score indicates more functional difficulty. The WHODAS has been used in a range of settings and in previous research in Nepal, including interventions that have focused on behavioral activation. Internal consistency for the WHODAS in the Chitwan population is alpha=.84 [48]. These markers will help in tracking participant improvement in daily functioning because of the support of providers and the use of technology.
4. Home Observation Measurement of the Environment (HOME): the HOME is a 45-item measure of the child’s exposure to stimulating interactions, environments, and emotional support [85,86]. The measure is comprised of 6 subscales including responsivity, acceptance, organization, learning materials, involvement, and variety and are elicited with a combination of rater observations during the home visit and direct elicitation of self-report from the parent. The HOME scale has been used extensively in South Asia [87-89] and was adapted to ensure appropriateness for the Nepali context.
5. Observation of Mother and Child Interaction (OMCI) [88,90-92]: the OMCI is an observational tool that was developed and used extensively in South Asia to assess mother-child interactions in a live-coded format. In its development, the tool had high interobserver reliability and was significantly correlated with the responsivity and involvement subscales of the HOME [91]. The tool measures 4 typical domains of maternal responsive behaviors (responsivity, emotional-affective support, support for infant attention, and language stimulation). The tool includes 18 items, with a range from 0 to 54, and higher scores indicate more positive interaction. Rather than standard assessments that require video recording in laboratory-like settings, this technique was adapted for more feasible and rapid use in low-income contexts. Instead of requiring a video, it uses a live coding framework. The OMCI is conducted by a trained female research assistant during the second or third home visit to the newly recruited mother. The mother is given soft toys appropriate for a Nepali infant (baby rattles, trumpet, and squeeze animals toys for 3- to 9-month-old babies; car, bigger rattles, and pinwheel for 6- to 12-month-old babies) and instructed to play with her child as she normally would for 5 minutes. After the first minute, the research assistant counts the instances in which a particular behavior occurs (eg, intrusive behaviors). In addition to the live coding, the interaction will be video recorded. We are using the OMCI in a qualitative manner to benchmark the technology findings, particularly related to verbal interactions and interaction quality (elicited through the episodic audio recording with subsequent machine learning) and therefore provide more reliable results of our findings. Thus, we expect that mothers with less verbal interaction (as recorded by the passive sensing domain—audio with conversations) and less consistent beacon proximity data (as determined by the passive sensing domains—time spent with and away from infant) will have lower OMCI scores on the corresponding items/domains (OMCI domains—verbal statements and communication).

##### Qualitative Data Collection

1. Key informant interviews will be conducted with each woman enrolled in component 2 within the first 3 days of passive data sensing data collection and again at end line (on the 14th day). The first key informant interview elicits the experience of adolescent mothers in relation to her pregnancy, childbirth, and depression. The interview also explores her perceptions and experiences of self-care, help seeking, and interpersonal relationships. This key informant interview is particularly helpful for our team to optimize the passive sensing data for the culture and context of its users. The second key informant interview will be conducted on the use of technology. Interviews will elicit several domains about the feasibility, acceptability, barriers, and facilitators of collecting passive sensing data.
2. Daily diary elicitation will be done by trained Research assistants with the mother recounting her activity; location; child’s activity, location, and caregiver; and who else was with her together on the hourly basis for a single day in the past week. It also elicits from the mother the time she awoke in the morning, went to bed in the evening and approximately when she fell asleep. Interruptions and triggers for waking up were also documented. The research assistant also asks the mother to self-evaluate her mood during the morning, afternoon, and evening in the previous day using 5 emoticons ranging from sad to happy. This activity allows us to triangulate the passive sensing data collected from the mother for accuracy.

#### Component 3

On the basis of the feedback from components 1 and 2, we will start the care monitoring phase (component 3) where we will provide the providers with the passive sensing data which will be incorporated during HAP sessions. With these data, the providers can provide tailored counseling sessions to the adolescent mothers with the risk of depression. We will develop the StandStrong app, which will be used to systematically visualize the passive sensing data. In the care monitoring phase, we will refer the depressed adolescent mothers recruited in the observational phase to HAP counseling sessions. We will provide the providers with a tablet with StandStrong app to access passive sensing data of the depressed mothers. The HAP providers can then provide tailored HAP sessions based on the case’s passive sensing data as visualized in the StandStrong app. In addition, we will conduct exploratory analyses on changes over time of the behaviors monitored by the passive sensing data.

##### Participants

We will use the same recruitment criteria for component 3 as was used for depressed mothers in component 2. Participants from component 3 will also be analyzed as cases in Component 2 using the first two weeks of their passive data collection. Therefore, we anticipate that some of the 20 depressed women recruited for component 2 will also be included in component 3.

##### Quantitative Data Collection

All the quantitative data collection used in component 2 will also be used for component 3. In addition, because component 3 involves a therapeutic intervention (HAP), the BDI is also ideal to measure changes in symptom severity over the course of implementation. The BDI will be administered weekly for approximately 6 to 8 weeks.

##### Qualitative Data Collection

Similar to component 2, for component 3, key informant interviews will be conducted with each woman within the first 3 days of passive data sensing data collection and again at end line (after approximately 5 weeks). This key informant interview is particularly helpful for our team to optimize the passive sensing data and the StandStrong app for the culture and context of its users. The second key informant interview will be conducted on the use of technology and the integration of passive sensing data in StandStrong app. Interviews will elicit several domains about the feasibility, acceptability, barriers, and facilitators of using and understanding the passive sensing data used for the intervention.

### Data Analysis

#### Qualitative Data Analysis

Informed consent will be documented from each participant. All qualitative interviews will be conducted in Nepali, transcribed verbatim (also in Nepali), and then translated into English, preserving culturally meaningful terms. Textual transcripts will be imported into qualitative data analysis software and systematically coded for the themes described above. Content analysis will be used to reduce, synthesize, and provide rich descriptions for StandStrong’s acceptability and feasibility, particularly to assess if, and how, the approach can be brought to a larger scale. We will systematically triangulate each passive data collection strategy with the appropriate qualitative and quantitative data (Table 3). The GPS and beacon proximity data will be triangulated with the daily diary elicitation. The Episodic Audio Recorder (EAR) output will be triangulated with the OMCI and HOME. Finally, the exit interview asks the mother to tell us if she thinks her data (seen in the StandStrong app) is accurate and asks her to interpret and describe her behaviors.

**Table 3:** An overview of the data collection methods and outcome measures.

Domain	Data type	Methods	Passive data	Measures	Components		
					I	II	III
Passive sensing data	Quantitative	GPS^a^ (mobile phone)	Movement	Amount of time at the house	—^b^	x^c^	x
Passive sensing data	Quantitative	GPS (mobile phone)	Movement	Time spent outside the house	—	x	x
Passive sensing data	Quantitative	Accelerometer (mobile phone)	Activity	Activity—time spent standing, walking, running.	—	x	x
Passive sensing data	Quantitative	Proximity beacon (proximity beacon)	Proximity	Time spent with the child	—	x	x
Passive sensing data	Quantitative	Proximity beacon (proximity beacon)	Proximity	Time spent away from child (self-care)	—	x	x
Passive sensing data	Quantitative	Proximity beacon (proximity beacon)	Proximity	The consistency of interaction between mother and child	—	x	x
Passive sensing data	Quantitative	Episodic audio recorder (mobile phone)	Audio with conversations	Social interaction (conversation)	—	x	x
Environment	Quantitative	Home Observation Measurement of the Environment inventory	N/A^d^	A 45-item tool to assess the home environment in terms of responsivity, acceptance, organization, learning materials, involvement, and variety	—	x	x
Environment	Quantitative	Observation of Mother-Child Interaction	N/A	An 18-item tool to assess the quality of interaction between mother and child.	—	x	x
Environment	Qualitative	Day in Life	N/A	An hour-by-hour description of participant’s activities over an average day (4 am to 10 pm) to record scheduled activities.	—	x	x
Feasibility, acceptability, and utility	Qualitative	Focus group discussion with community advisory board members	N/A	—	x	x	x
Feasibility, acceptability, and utility	Qualitative	Key informant interview with adolescent mothers on motherhood	N/A	—	—	x	x
Feasibility, acceptability, and utility	Qualitative	Key informant interview with providers	N/A	—	—	—	x
Feasibility, acceptability, and utility	Qualitative	Key informant interview with adolescent mothers on technology	N/A	—	—	x	x

^a^GPS: Global Positioning System.

^b^Passive data will not be collected.

^c^Passive data will be collected.

^d^N/A: not applicable.

#### Quantitative Data Analysis

**Figure 6:**
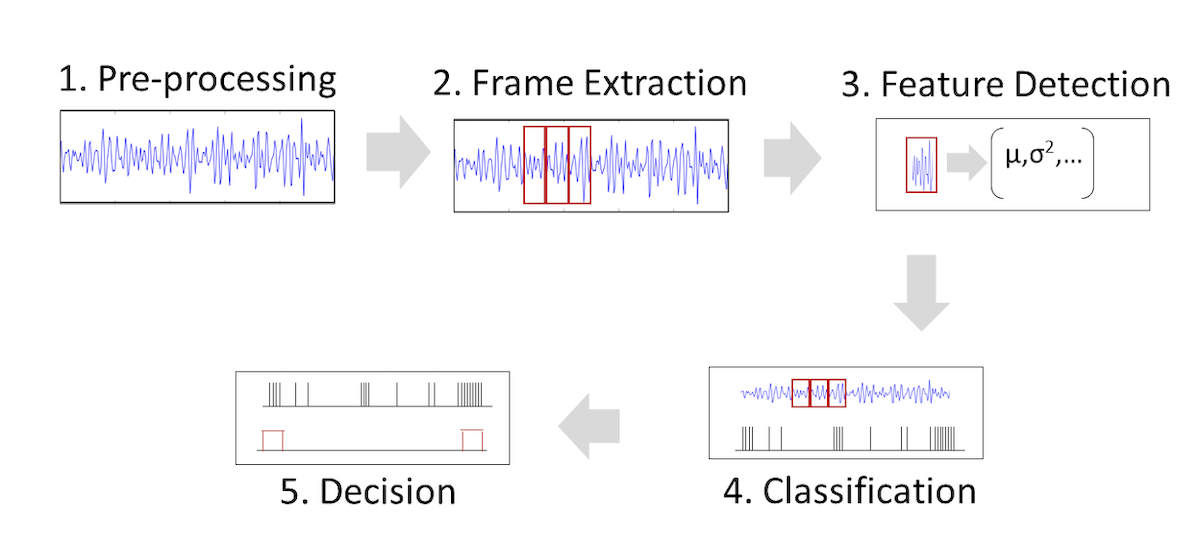
Analytic pipeline for sensor data.

This project will be dealing with quantitative unstructured data produced passively by a range of sensors. These sensors include proximity, GPS location, and episodic audio recordings. Working and analyzing these data are complex, and many of the analytic methods are still experimental and evolving. There is however an agreed-upon analytic pipeline that will be used for all sensor data collected in this study (Figure 6).

1. Preprocessing: the first step in the analytic plan is to process the raw data files and prepare them for the subsequent steps. During this stage, data are cleaned, and missing values and outliers are addressed through mean replacement or other similar techniques. Data are restructured from a raw comma-separated values into a data frame that can be easily manipulated in Python using a machine learning package such as scikit-learn or Tensorflow.
2. Frame extraction: as the sensor data are time stamped and the feature of interest (such as a spoken word) occurs over a number of seconds, data need to be split into segments using a sliding time window. The literature suggests appropriate window lengths for a different feature. For example, a laugh may last 5 seconds while a cough usually occurs over a period of only a second. This window is then passed over the raw data splitting an uninterrupted data signal into segments of homogeneous content.
3. Feature detection: feature extraction is an important analysis stage. During this stage, we will extract a set of data elements that provide representative characteristics of the frame. The aim of this analysis is to form a feature vector for each frame that can serve as an input to the classifier. Feature extraction can also be viewed as data rate reduction procedure and allows the classification analysis to be based on a reduced set of feature vectors. We will extract 2 types of features from the audio sensor data collected in this study, namely temporal and spectral features. We plan to use Fast Fourier Transform to extract temporal features include zero-crossing rate, short-time energy, energy entropy, root mean square, and autocorrelation. Spectral features of the raw sensor data will be extracted using mel-frequency cepstrum coefficients.
4. Classification: classifiers can be divided into 2 groups—classifiers that use supervised learning (supervised classification) and unsupervised learning (unsupervised classification). In supervised classification, examples of the correct classification are provided to the classifier. On the basis of these examples, which are commonly termed as training samples, the classifier then learns how to assign an unseen feature vector to a correct class. Examples of supervised classifications include Hidden Markov Model, Gaussian Mixture Models, K-Nearest Neighbor, and Support Vector Machines. In unsupervised classification or clustering, there is neither explicit teacher nor training samples. This study will make use of supervised learning with data taken from the early stages used to build and train a model capable of classifying unseen sensor data.
5. Decision making: decision making is the final stage in the analytic pipeline. In this study, we will compute simple summary statistics of interest that can be fed back to participants in a meaningful way. These statistics will include percent time with child percent verbal stimulation of child and percent of distressed vocalizations.

### Data Validation

We aim to validate the sensing data using qualitative and quantitative data collected from the study tools. Our sensing data (activity, GPS, and proximity) will be validated using qualitative (Day in Life) and quantitative (HOME, OMCI, WHODAS, BDI, and PHQ) tools. Our team has included several other measures to benchmark the passive sensing data particularly because the novel technology has few markers of precision and sensitivity. Finally, we expect that mothers with fewer stimulating occurrences (described above with the EAR) and mothers with prolonged time with their infant (proximity) to have lower HOME and OMCI scores.

### Ethical Approval

The study has been granted ethical approval by the Nepal Health Research Council (327/2018) and George Washington University Institutional Review Board (#051845).

## Results

The study is currently underway, and recruitment for participants is open. A community advisory board for the study has been formed. The community advisory board reviewed the objectives and key components of the protocol. The community advisory board also reviewed the technology to be used by observing videos demonstrating the technology [70] and through a live demonstration of the technology. The community advisory board provided input on methods of recruitment and how best to explain the study to adolescent mothers and their in-laws with whom they reside. In addition, based on feedback from the community advisory board, local research team, and initial participants, the final mental health evaluation tools (PHQ-9 for screening and BDI for symptom evaluation) were selected.

## Discussion

Given the burden of untreated depression globally, especially among adolescents in LMIC, there is a need to identify approaches to improve identification of who will benefit from psychological services and to make those services more effective. Passive sensing data have the potential to improve accuracy of detection and enhance personalization of services, thus leading to greater effectiveness and sustainability of mood and behavioral changes. Upon completion of this study, we will have greater knowledge of what is feasible and acceptable for the use of mobile technology to collect passive sensing data among adolescent mothers with infants. We also will have results of exploratory analyses comparing depressed and nondepressed mothers on passive sensing data outcomes to determine what domains most accurately identify depression. Moreover, at the culmination of the study, there will be a mobile platform for nonspecialists and the mothers they are treating. Successful completion of this study will thus advance knowledge about postpartum depression and establish new applications of technology and data to improve the lives of adolescent mothers. At a policy level, Nepal formulated a National Electronic Health Strategy in 2017 and is committed to integrating electronic and mobile technologies to increase effectiveness and access to health care services [93]. Given that projects in Nepal such as PRIME have successfully integrated nonspecialists in mental health care service delivery [48], the findings from this pilot study can advance the benefits of mobile technology for psychosocial counseling services delivered by the nonspecialists.

In addition to the service delivery benefits in LMICs, the methods of the proposed study have implications for mental health interventions in the high-resource settings as well. In high-income countries, there has been an explosion of mHealth apps addressing mental health and psychological well-being [94]. However, most of these apps are self-directed and do not involve a therapist. A recent review of cognitive behavioral therapies found that unguided self-help, such as using an app without also having a human component, does not have comparable benefit to individual, group, phone-based, and guided self-help that includes a human interaction [95]. One of the challenges is that most therapists in high-income countries are not trained on how to incorporate mHealth data into existing therapies [94-96]. The StandStrong app provides a framework for therapists to work with clients, measure progress, and identify opportunities for therapeutic goals. To date, apps for physical health condition (eg, diabetes and cardiovascular disorders) have demonstrated added value when the health professionals are engaging with the app alongside patients [97,98]. Similarly, the StandStrong app could provide a platform for greater collaboration between clients and providers, rather than an interface that is only used by clients. Moreover, within the field of mental health, most outcome and process measurements continue to rely on self-reports. The United States National Institute of Mental Health has called for more objective measures of what happens in mental health interventions [99]. This study advances work in objective measurement by tracking behavioral changes over the course of an intervention. This will shed light on what aspects of behavioral change are linked with improved mood and well-being.

## Appendix

Multimedia Appendix 1 StandStrong app demo.

## Abbreviations

BDI: Beck Depression Inventory
EAR: episodic audio recorder
EBM: electronic behavior monitoring
FCC: Federal Communications Commission
FDA: Food and Drug Administration
GPS: Global Positioning System
HAP: Healthy Activity Program
HOME: Home Observation Measurement of the Environment
LMIC: low- and middle-income countries
mHealth: mobile health
OMCI: observation of mother-child interaction
PHQ-9: Patient Health Questionnaire
PRIME: Program for Improving Mental Health Care
RSSI: received signal strength indication
StandStrong: Sensing Technologies for Maternal Depression Treatment in Low Resource Settings
WHODAS: World Health Organization Disability Assessment Scale
