# Correction: Wearable Digital Sensors to Identify Risks of Postpartum Depression and Personalize Psychological Treatment for Adolescent Mothers: Protocol for a Mixed Methods Exploratory Study in Rural Nepal

**DOI:** 10.2196/16837

**Published:** 2019-10-30

**Authors:** Anubhuti Poudyal, Alastair van Heerden, Ashley Hagaman, Sujen Man Maharjan, Prabin Byanjankar, Prasansa Subba, Brandon A Kohrt

**Affiliations:** 1 Division of Global Mental Health Department of Psychiatry and Behavioral Sciences George Washington School of Medicine and Health Sciences Washington, DC United States; 2 Human and Social Development Human Sciences Research Council Pietermaritzburg South Africa; 3 Medical Research Council/Wits Developmental Pathways for Health Research Unit Department of Paediatrics, Faculty of Health Sciences University of the Witwatersrand Johannesburg South Africa; 4 Department of Social and Behavioral Sciences Yale School of Public Health Yale University New Haven, CT United States; 5 Center for Methods in Implementation and Prevention Science Yale University New Haven, CT United States; 6 Transcultural Psychosocial Organization Nepal Kathmandu Nepal; 7 United Mission to Nepal Kathmandu Nepal

In “Wearable Digital Sensors to Identify Risks of Postpartum Depression and Personalize Psychological Treatment for Adolescent Mothers: Protocol for a Mixed Methods Exploratory Study in Rural Nepal” by Poudyal et al (JMIR Res Protoc 2019;8(9):e14734), a minor error in the typesetting stage of publication resulted in the International Registered Report Identifier (IRRID) not being included in the final version of the article.

The IRRID “DERR1-10.2196/14734” has now been added to the paper.

The correction will appear in the online version of the paper on the JMIR website on October 30, 2019, together with the publication of this correction notice. Because this was made after submission to PubMed, PubMed Central, and other full-text repositories, the corrected article has also been resubmitted to those repositories.

